# A biochemical marker panel in MRI-proven hyperacute ischemic stroke-a prospective study

**DOI:** 10.1186/1471-2377-12-14

**Published:** 2012-03-08

**Authors:** Carolin Knauer, Katharina Knauer, Susanne Müller, Albert C Ludolph, Dietmar Bengel, Hans P Müller, Roman Huber

**Affiliations:** 1 Department of Neurology, University of Ulm, Oberer Eselsberg 45, 89075 Ulm, Germany; 2 Department of Neurology, Oberschwabenklinik, Elisabethenstraße 15, 88212 Ravensburg, Germany

**Keywords:** Stroke, Biochemical marker, Brain natriuretic peptide, D-dimers, Matrix-metalloproteinase-9

## Abstract

**Background:**

Computer tomography (CT) is still the fastest and most robust technique to rule out ICH in acute stroke. However CT-sensitivity for detection of ischemic stroke in the hyperacute phase is still relatively low. Moreover the validity of pure clinical judgment is diminished by several stroke imitating diseases (mimics). The "Triage^® ^Stroke Panel", a biochemical multimarker assay, detects Brain Natriuretic Peptide (BNP), D-Dimers (DD), Matrix-Metalloproteinase-9 (MMP-9), and S100B protein and promptly generates a Multimarkerindex of these values (MMX). This index has been licensed for diagnostic purposes as it might increase the validity of the clinical diagnosis to differentiate between stroke imitating diseases and true ischemic strokes. Our aim was to prove whether the panel is a reliable indicating device for the diagnosis of ischemic stroke in a time window of 6 h to fasten the pre- and intrahospital pathway to fibrinolysis.

**Methods:**

We investigated all consecutive patients admitted to our stroke unit during a time period of 5 months. Only patients with clinical investigation, blood sample collection and MRI within six hours from symptom onset were included. Values of biochemical markers were analyzed according to the results of diffusion weighted MR-imaging. In addition MMX-values in ischemic strokes were correlated with the TOAST-criteria. For statistical analysis the SAS Analyst software was used. Correlation coefficients were analyzed and comparison tests for two or more groups were performed. Statistical significance was assumed in case of *p *< 0.05. Finally a ROC-analysis was performed for the MMX-Index.

**Results:**

In total 174 patients were included into this study (n = 100 strokes, n = 49 mimics, n = 25 transitoric ischemic attacks). In patients with ischemic strokes the mean NIHSS was 7.6 ± 6.2, while the mean DWI-lesion volume was 20.6 ml (range 186.9 to 4.2 ml). According to the MMX or the individual markers there was no statistically significant difference between the group of ischemic strokes and the group of mimics. Moreover the correlation of the index and the DWI-lesion-volume was poor (*p *= 0.2).

**Conclusions:**

In our setting of acute MRI-proven ischemic stroke the used multimarker-assay (Triage^® ^Stroke Panel) was not of diagnostic validity. We do not recommend to perform this assay as this might lead to a unjustified time delay.

## Background

Stroke represents the 2nd leading cause of death and the most common cause of morbidity in industrialized countries. Although systemic fibrinolysis has been proven to be an effective therapy even in industrialized countries only the minority of all ischemic stroke patients receives rtPA therapy [1,2]. Therefore, acute stroke therapy remains one of the essential challenges of Neurology. In case of acute stroke it's important to apply immediately reliable diagnostic tools to start treatment as soon as possible as the time to recanalization is a very substantial predictor of the clinical outcome [3]. Acute stroke-imaging is still predominantly based on CT although detection of ischemic lesions at this time point is often difficult as early ischemic signs are only present in one third to one half of the cases [4]. Even with sophisticated procedures like perfusion CT it is not possible to yield a higher sensitivity especially in case of non-territorial infarctions [5]. In contrast MRI provides a very high sensitivity and specificity for detection of ischemic strokes even in the hyperacute stage [6-8].

However a comprehensive availability of MRI has not been achieved yet. Thus the diagnosis of ischemic stroke in most cases is still based on clinical estimation after ruling out intracerebral hemorrhage (ICH) by CT. However several other diseases like seizures or migraine make the correct diagnosis difficult. To enhance the clinical sensitivity and specificity several investigations on biochemical markers were performed. According to a significant increase of markers of inflammation, thrombosis and cellular death as well as myelin damage within 24 h from stroke onset a point-of-care immunoassay (Triage Stroke Panel^®^) has been developed. This system rapidly analyses Brain Natriuretic peptide (BNP), D-Dimers (DD), Matrix Metalloproteinase-9 (MMP-9) and S 100 B protein to estimate-together with a Multimarkerindex (MMX)-the probability of stroke. The aim of our investigation was to prove, whether this panel could enhance the diagnostic reliability and differentiate between ischemic stroke patients and patients with stroke imitating diseases (mimics).

## Methods

All patients consecutively admitted over a time period of 5 months to the stroke unit of the University Hospital of Ulm with the tentative diagnosis of an acute stroke were investigated. Patients were only included into the study if they were admitted in a maximum time frame of 6 h from symptom onset. Clinical investigation and collection of blood samples were performed immediately after admission. This procedure was directly followed by a typical standard MRI-based stroke imaging protocol (DWI, T2*w, TOF-MRA, FLAIR, T2w, T1w, PWI). Patients who did not receive a MRI, e.g. due to contraindications to magnetic resonance imaging or for technical reasons were not included.

Finally patients were divided into three groups: group I consisted of ischemic stroke patients according to the judgment of the treating neurologist confirmed by a typical DWI-lesion. This group included also patients that were clinically classified as transitoric ischemic attack (TIA), but demonstrated an acute DWI-lesion correlating with the neurological deficit. In the absence of a typical DWI-lesion these patients were categorized in the classical way as TIA and included in group II. All other patients were included into the group III with patients with stroke imitating diseases (mimics).

In group I the volume of the ischemic lesions was measured by determining the DWI-lesion size using an in-house developed volumetric software (TIFT). The analysis of the biochemical markers (BNP, D-Dimers, MMP-9, S 100 B, MMX) was done by Sandwich-Fluorescence-Immunoassay-Technology of the so-called Triage^® ^Stroke Panel using blood from an EDTA sample taken within 15 min after admission. All stroke patients got the generally performed diagnostic investigations including sonography of the extracranial and intracranial arteries, ECG, long-term ECG and transthoracal echocardiography. Transoesophageal echocardiography was performed on an individual base. The TOAST classification was used to define the stroke etiology [9]. Clinically the severity of the neurological deficit was assessed using the National Institute of Health Stroke Scale (NIHSS). Clinical development was evaluated by comparing the baseline NIHSS score ad admission with a repeated investigation measured 7 days after stroke or at discharge from the stroke unit. Statistics were performed by the SAS Analyst software. General characterization of patients was done by a descriptive analysis. The specifitity and sensitivity of the MMX as well as of the single parameters of the panel were calculated by comparing the results between group I and III performing a ROC -Analysis. To rule out any uncertainty we excluded the TIA group from this analysis. In addition the results of the biochemical markers in the stroke group were correlated with the size of the respective acute DWI-restrictions in the MRI. For the correlation analysis Spearman correlation coefficients (*p*) and scatter plots were used. For pointing out differences between groups we performed the Fisher exact test (2 variables) and *χ*^2^-test (> 2 variables) for non-continuous variables and the Wilcoxon- (2 variables) and Kruskal-Wallis-Test (> 2 variables) for continuous variables. Statistical significance was assumed in case of *p *< 0.05. Finally a ROC-analysis was performed for the MMX-Index.

The ethics committee of the University of ULM approved the study. Written informed consent was obtained by all included patients or their legal guardian.

## Results

In total 174 patients were included. The cohort consisted of 100 ischemic stroke patients (group I) with a median age of 73 years (range 18-97 years), 25 patients with TIAs (group II) with a median age of 74 years (range 40-88 years) and 49 mimics patients (group III) with a median age of 65 years (range 22-93 years). Demographic as well as clinical characteristics of patients are shown in Table 1. Beside a higher incidence of atrial fibrillation in the group of ischemic stroke patients there were no significant differences between the three groups in terms of demographic characteristics and vascular risk factors.

**Table 1:** Demographic characteristics and vascular risk factors

	Strokes	TIAs	Mimics	*p*-value
Numers (n)	100	25	49	

Age (years): median (range)	73 (18;97)	74 (40;88)	65 (22;93)	0.13

Gender: no of males (%)	52 (52)	11 (44)	27 (55)	0.62

Hypertension: n (%)	76 (76)	21 (84)	31 (63)	0.11

Diabetes: n (%)	32 (32)	5 (20)	9 (18)	0.15

Smokers: n (%)	14 (14)	5 (20)	6 (12)	0.66

Dyslipidemia: n (%)	44 (44)	13 (52)	14 (29)	0.09

Atrial fibrillation: n (%)	35 (35)	3 (12)	7 (14)	0.006*

Admission after the first symptoms was within 1 h in 9% of the patients, between 1 h and 3 h in 56% and after 3 up to 6 h in 35% of all patients. The mean admission time was 3.03 ± 1.74 h after the first symptoms. Patients in the mimics group were suffering from migraine with aura (n = 4), other types of headache (n = 3), seizures with concomitant postictual paresis (n = 12), dizziness (n = 7), syncopes (n = 8), hypertensive encephalopathy (n = 1), idiopathic paresis of the facial nerve (n = 1), transient global amnesia (n = 2) and other diagnosis (n = 11). According to the TOAST-criteria 35% of the ischemic stroke patients suffered from cardio-embolic strokes, 39% from stroke of thrombembolic origin and only 3% were judged to have microangiopathic strokes. In 4% other, rare causes of stroke have been found, while in 19% the origin of the stroke remained unclear or was possibly due to more than one single cause. 76% of the ischemic strokes were localized in the anterior circulation territory (70% in MCA territory) and 23% in the vertebrobasilar territory (10% in PCA territory, 10% in the brain stem, 10% in the PICA territory). We quantified the acute lesion size by an in-house developed software measuring the acute DWI-lesion size on admission, which means immediately after collecting the blood sample for the biochemical analysis. The mean lesion size was 20.6 ml ranging from 4.2 to 186.9 ml. Clinically the mean NIHSS was 6 (median; range 0-25) at admission and 4 (median; range 0-19) at discharge. There was a significant correlation of the ischemic lesion size and the clinical presentation of strokes on admission as well as 7 days later (or at discharge) (Spearman's *p *= 0.57 and *p *= 0.47 respectively).

**Figure 1:**
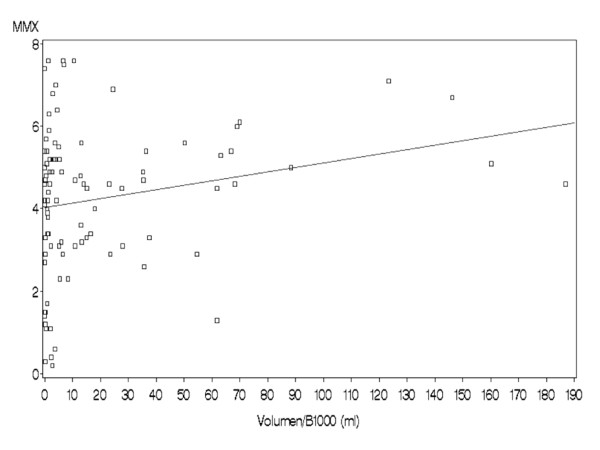
**Comparison of the DWI-lesion size and the MMX-values**. No significant correlation was obtained.

The triage stroke panel measures BNP, DD, MMP-9, S100 B and generates the Multimarkerindex of these values (MMX). In our investigation this index did not differ statistically significantly between the both groups of mimics and strokes (mean 4.2 ± 1.7 vs. 3.6 ± 2.0, n.s.). Moreover not even a modest correlation was found between the DWI-lesion size and the MMX-value (*p *= 0.2, see Figure 1: Comparison of the DWI-lesion size and the MMX-values. No significant correlation was obtained). According the time window from symptom onset MMX did not differ statistically significant between a time frame of 3 h and 6 h from symptom onset (median 4,5 and 4,6; n.s.).

The two most reasonable MMX cut-offs (2.3 and 2.5) which we could derive from our own dataset reached indeed sensitivities of 86% and 84%. However, the corresponding specificities were only 33% and 35%. For the officially recommended cut-offs at 1.3 and 5.9 we obtained either a high sensitivity of 92% with a low specificity of 14% or a good specificity of 86% and a poor sensitivity of 14% respectively. In general no cut-off value with a clinically useful sensitivity and specificity could be found (see Table 2)

**Table 2:** Diagnostic quality of MMX at different cut-off values

MMX cut-off	Sensitivity	Specificity
1.3	92%	14%

2.3	86%	33%

2.5	84%	35%

5.9	15%	86%

7.3	5%	94%

**Figure 2:**
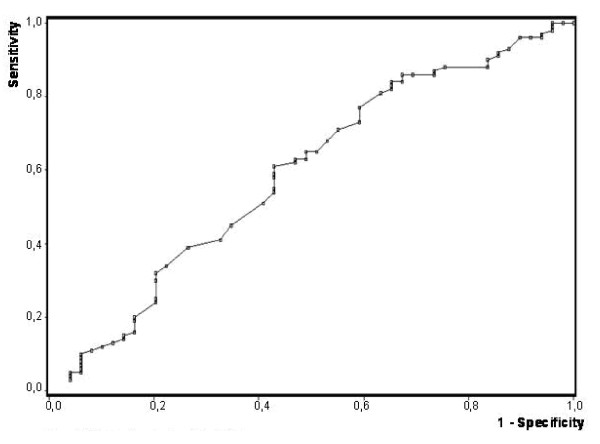
**ROC-Analysis of the MMX-Value with a poor AUC-Value of 0,59**.

Consequently the ROC analysis for MMX showed a low discriminatory power with an AUC-value of 0.59 (see Figure 2: ROC-Analysis of the MMX-Value with a poor AUC-Value of 0,59).

Similar results were obtained, when we performed statistical analysis on the correlation of the individual markers of the panel and the DWI-lesion-size. In our group nearly 98% (n = 170) of the patients did not reach the lower limit of the testing range for S100 B. Thus no statistics were performed for this individual item. For MMP-9 and D-Dimers no statistically significant differences were obtained between the two groups of mimics and strokes. Only measuring BNP-levels led to a significant difference between these both groups (median 64.5 in strokes vs. 19.3 in mimics *p *= 0,01, see Table 3)

**Table 3:** Median of the MMX and its individual Markers in the stroke and mimics group.

	Strokes	Mimics		Correlation to V_DWI _Spearman's ρ
	Median	Median	P	
**MMX**	4.6	3.5	0.07	0.2

**BNP (ng/ml)**	64.45	19.3	0.01*	0.09

**D-Dimers (ng/ml)**	675	322	0.10	0.09

**MMP 9 (ng/ml)**	78.9	101	0.66	0.06

In addition the correlation of these markers with the DWI-lesion size (VDWI) is given

**Figure 3:**
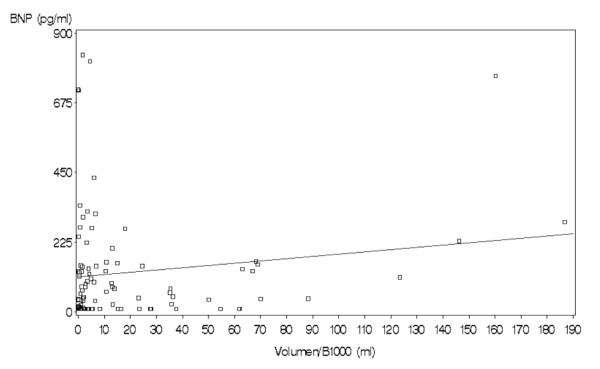
**Comparison of the DWI-lesion size and the BNP-values**. No significant correlation was obtained.

Further subgroup analysis yielded a strong significant trend (*p *= 0,002) with higher BNP-values in case of cardioembolic stroke according to the TOAST classification compared to other stroke etiologies (median 131 vs. 19,8). When we excluded patients with a supposed cardiogenic etiology from the stroke group, the difference between the other TOAST-groups to the mimics in terms of BNP did not longer stay significant. In addition, we didn't find a strong correlation with the DWI-lesion volume neither for the BNP-values nor for any other biochemical marker (*p *= 0,09, see Figure 3: Comparison of the DWI-lesion size and the BNP-values. No significant correlation was obtained).

## Discussion

An early and valid diagnosis expedites the way to fibrinolysis in ischemic stroke and excludes stroke imitating diseases from the potentially dangerous therapeutic intervention. To improve the reliability of the clinical diagnosis the Triage^® ^Stroke Panel has been developed. A recent investigation proved whether this multimarker assay could increase the diagnostic sensitivity of a clinical prehospital stroke scale in-between the first 24 h after the initial symptoms [10]. We wanted to prove the diagnostic sensitivity and specificity of this test solely in the therapeutically relevant time window up to six hours. To exclude any clinical uncertainty we compared the biomarker index as well as its individual parameters with the MRI results of ischemic stroke patients and stroke imitating diseases. In case of acute DWI-lesions patients clinically rated as TIAs were classified as ischemic strokes according to their greater risk of recurrent cerebrovascular events [11]. The group of TIA patients without DWI-lesions was not included in the statistical calculation of sensitivity and specificity in order to rule out any diagnostic uncertainty. Furthermore to avoid any selection bias patients were included consequently after the admission to our hospital. The group of ischemic strokes consisted of a quite common spectrum of older patients with typical stroke patterns, e.g. stroke of the anterior circulation of mostly thromboembolic origin. The only demographic difference was a higher proportion of patients with atrial fibrillation in the group of ischemic strokes compared to the group of TIA's and mimics. Consistently according to the TOAST-criteria the treating physicians estimated a percentage of cardiogenic strokes higher than usual, while only a few strokes were judged as small vessel disease. The latter is possibly due to the short time window and the tendency to later admissions of lacunar strokes with minor clinical deficits [12]. In contrast to earlier investigations [10,13,14] in our cohort the multimarker-index or its individual items were not able to differentiate between the groups of ischemic strokes and mimics. This is possibly due to several reasons. First in our cohort ischemic stroke was diagnosed according to a combination of the clinical judgment and MR-imaging, which is clearly superior to a diagnosis based on pure clinical judgment or on a combination of clinical judgment and CT-imaging. This is especially true in case of smaller ischemic lesions with clinical syndromes that do not directly give direction to the correct diagnosis. Therefore based on MRI we possibly included patients who otherwise would not have been classified as ischemic stroke. In contrast so called MRI-negative ischemic strokes might led to the exclusion of patients which would have been classified as ischemic stroke based upon clinical judgment. However neither the imaging parameters of our patients nor the clinical severity of disease give rise to suchlike considerations. Both the DWI-lesion size [15,16] as well as the mean NIHSS on admission [10] were quite comparable to other investigations. The one rather critical difference certainly is the narrow time window, as we only included patients up to 6 h after the onset of the first symptoms. Although a lot of biomarkers have been shown to rise after an ischemic stroke, most of them increase with a considerably longer delay. While S 100 B seems to peak after more than one day [17], MMP 9 seems to rise within the first eight hours after stroke reaching its maximum after 24 h [18]. This lag of time explains the missing difference between the group of ischemic strokes and stroke imitating diseases for S100 B and MMP-9 in our cohort most likely. The solely marker significantly heightened in the ischemic stroke population compared to the mimics group at this early time point was BNP. However this effect was caused by the patients assumedly suffering from cardiogenic stroke. These results are in concordance with several other investigations, which demonstrated an augmented expression of BNP predominantly in stroke of cardiac origin [19,20], while other etiologies did not necessarily demonstrate this elevation [21-23].

Moreover there was no correlation between these single parameters (or the MMX-value) and the ischemic lesion size measured by the acute DWI-lesion volume. Although there are several reports about shrinking DWI-deficits, DWI-lesions at least approximately represent the infarct core [24]. From a pathophysiological point of view a biomarker for identifying ischemic strokes should depend on the amount of irreversible damaged brain tissue. One candidate marker indicating neuronal destruction would be NSE (Neuronspecific Enolase) [25,26].

As we missed to demonstrate a suchlike relationship and the MMX Cut -off points reached only a weak and clinically not reasonably sensitivity and specificity we couldn't demonstrate a general diagnostic value for the investigated biochemical markers in our setting of hyperacute ischemic stroke.

## Conclusions

Neither one marker nor combination of all markers is of significant benefit in acute stroke diagnostics. DWI-MRI is still the procedure with the highest diagnostic quality in case of acute cerebral ischemia. Nevertheless further investigation of biochemical markers might lead to higher diagnostic quality in acute stroke diagnosis, e.g. in the differentiation of etiological subgroups.

## Competing interests

The authors declare that they have no competing interests.

## Authors' contributions

CK was involved in composing the study design, the data collection, statistical analysis and in drafting the manuscript. KK contributed to the interpretation of the results and to the critical revision of the article. SM contributed to the revision of the manuscript. ACL contributed to the critical discussion of the study design and concept and revision of the article. DB contributed to the revision of the manuscript. HPM developed the volumetric software (TIFT). RH was involved in the conception of the study design, the interpretation of the results and in drafting the manuscript. All authors read and approved the final manuscript.

## Pre-publication history

The pre-publication history for this paper can be accessed here:

http://www.biomedcentral.com/1471-2377/12/14/prepub

## References

[B1] Weimar C Kraywinkel K Maschke M Diener HC German Stroke SC Intravenous thrombolysis in German stroke units before and after regulatory approval of recombinant tissue plasminogen activator Cerebrovasc Dis 2006 22 5-6 429 431 10.1159/000094995 16912477

[B2] Hacke W Donnan G Fieschi C Kaste M Association of outcome with early stroke treatment: pooled analysis of ATLANTIS, ECASS, and NINDS rt-PA stroke trials Lancet 2004 363 9411 768 774 15016487 10.1016/S0140-6736(04)15692-4

[B3] Hacke W Brott T Caplan L Meier D Fieschi C von KR Donnan G Heiss WD Wahlgren NG Spranger M Thrombolysis in acute ischemic stroke: controlled trials and clinical experience. [Review] [12 refs] Neurol 1999 53 7 Suppl 4 S3 14 10532643

[B4] de Camargo EC Sa Koroshetz WJ Neuroimaging of ischemia and infarction. [Review] [97 refs] NeuroRx 2005 2 2 265 276 10.1602/neurorx.2.2.265 15897950 PMC1064991

[B5] Mejdoubi M Calviere L Dumas H [Value of CT perfusion for the diagnosis of early middle cerebral artery stroke] J Radiol 2010 91 5 Pt 1 555 560 20657354 10.1016/s0221-0363(10)70087-1

[B6] Davis DP Robertson T Imbesi SG Davis DP Robertson T Imbesi SG Diffusion-weighted magnetic resonance imaging versus computed tomography in the diagnosis of acute ischemic stroke J Emerg Med 2006 31 3 269 277 10.1016/j.jemermed.2005.10.003 16982360

[B7] von Kummer R Dzialowski I von Kummer R Dzialowski I MRI versus CT in acute stroke Lancet 2007 369 9570 1341 1342 author reply 1342 17448808 10.1016/S0140-6736(07)60621-7

[B8] Brazzelli M Sandercock PA Chappell FM Celani MG Righetti E Arestis N Wardlaw JM Deeks JJ Brazzelli M Sandercock PA Magnetic resonance imaging versus computed tomography for detection of acute vascular lesions in patients presenting with stroke symptoms Cochrane Database Syst Rev 2009 4 CD007424 19821415 10.1002/14651858.CD007424.pub2 PMC13436845

[B9] Adams HP Jr Bendixen BH Kappelle LJ Biller J Love BB Gordon DL Marsh EE III Classification of subtype of acute ischemic stroke. Definitions for use in a multicenter clinical trial. TOAST. Trial of Org 10172 in Acute Stroke Treatment Stroke 1993 24 1 35 41 10.1161/01.STR.24.1.35 7678184

[B10] Vanni S Polidori G Pepe G Chiarlone M Albani A Pagnanelli A Grifoni S Use of Biomarkers in Triage of Patients with Suspected Stroke J Emerg Med 2009 10.1016/j.jemermed.2008.09.028 19217237

[B11] Prabhakaran S Chong JY Sacco RL Prabhakaran S Chong JY Sacco RL Impact of abnormal diffusion-weighted imaging results on short-term outcome following transient ischemic attack Arch Neurol 2007 64 8 1105 1109 10.1001/archneur.64.8.1105 17698700

[B12] Qureshi AI Kirmani JF Sayed MA Safdar A Ahmed S Ferguson R Hershey LA Qazi KJ Buffalo Metropolitan A, Erie County Stroke Study G: Time to hospital arrival, use of thrombolytics, and in-hospital outcomes in ischemic stroke Neurol 2005 64 12 2115 2120 10.1212/01.WNL.0000165951.03373.25 15985583

[B13] Kim MH Kang SY Kim MC Lee WI Kim MH Kang SY Kim MC Lee WI Plasma biomarkers in the diagnosis of acute ischemic stroke Annals of Clinical & Laboratory Science 2010 40 4 336 341 20947807

[B14] Sibon I Rouanet F Meissner W Orgogozo JM Use of the Triage Stroke Panel in a neurologic emergency service American Journal of Emergency Medicine 2009 27 5 558 562 10.1016/j.ajem.2008.05.001 19497461

[B15] Rivers CS Wardlaw JM Armitage PA Bastin ME Carpenter TK Cvoro V Hand PJ Dennis MS Do acute diffusion- and perfusion-weighted MRI lesions identify final infarct volume in ischemic stroke? Stroke 2006 37 1 98 104 16322499 10.1161/01.STR.0000195197.66606.bb

[B16] Thijs VN Lansberg MG Beaulieu C Marks MP Moseley ME Albers GW Is early ischemic lesion volume on diffusion-weighted imaging an independent predictor of stroke outcome? A multivariable analysis Stroke 2000 31 11 2597 2602 10.1161/01.STR.31.11.2597 11062281

[B17] Nash DL Bellolio MF Stead LG Nash DL Bellolio MF Stead LG S100 as a marker of acute brain ischemia: a systematic review Neurocritical Care 2008 8 2 301 307 10.1007/s12028-007-9019-x 17968519

[B18] Ramos-Fernandez M Bellolio MF Stead LG Ramos-Fernandez M Bellolio MF Stead LG Matrix metalloproteinase-9 as a marker for acute ischemic stroke: a systematic review Journal of Stroke & Cerebrovascular Diseases 2011 20 1 47 54 10.1016/j.jstrokecerebrovasdis.2009.10.008 21044610

[B19] Montaner J Perea-Gainza M Delgado P Ribo M Chacon P Rosell A Quintana M Palacios ME Molina CA varez-Sabin J Etiologic diagnosis of ischemic stroke subtypes with plasma biomarkers Stroke 2008 39 8 2280 7 10.1161/STROKEAHA.107.505354 18535284

[B20] Rodriguez-Yanez M Castellanos M Blanco M Garcia MM Nombela F Serena J Leira R Lizasoain I Davalos A Castillo J New-onset hypertension and inflammatory response/poor outcome in acute ischemic stroke Neurology 2006 67 11 1973 1978 10.1212/01.wnl.0000247064.53130.91 17159103

[B21] Giannakoulas G Hatzitolios A Karvounis H Koliakos G Charitandi A Dimitroulas T Savopoulos C Tsirogianni E Louridas G N-terminal pro-brain natriuretic peptide levels are elevated in patients with acute ischemic stroke Angiology 2005 56 6 723 730 10.1177/000331970505600610 16327949

[B22] Iltumur K Karabulut A Apak I Aluclu U Ariturk Z Toprak N Elevated plasma N-terminal pro-brain natriuretic peptide levels in acute ischemic stroke Am Hear J 2006 151 5 1115 1122 10.1016/j.ahj.2005.05.022 16644347

[B23] Tomita H Metoki N Saitoh G Ashitate T Echizen T Katoh C Fukuda M Yasujima M Osanai T Okumura K Elevated plasma brain natriuretic peptide levels independent of heart disease in acute ischemic stroke: correlation with stroke severity Hypertension Research-Clinical & Experimental 2008 31 9 1695 1702 10.1291/hypres.31.1695 18971547

[B24] Chemmanam T Campbell BC Christensen S Nagakane Y Desmond PM Bladin CF Parsons MW Levi CR Barber PA Donnan GA Ischemic diffusion lesion reversal is uncommon and rarely alters perfusion-diffusion mismatch Neurology 75 12 1040 1047 20720188 10.1212/WNL.0b013e3181f39ab6

[B25] Kamchatov PR Ruleva NI Dugin SF Buriachkovskaia LI Chugunov AV Mikhailova NA Basse DA [Neurospecific proteins and autoantibodies in serum of patients with acute ischemic stroke]. [Russian] Zhurnal Nevrologii i Psikhiatrii Imeni SSKorsakova 2009 109 5 Suppl 2 69 72 19894304

[B26] Jauch EC Lindsell C Broderick J Fagan SC Tilley BC Levine SR Group Nr-PSS Association of serial biochemical markers with acute ischemic stroke: the National Institute of Neurological Disorders and Stroke recombinant tissue plasminogen activator Stroke Study Stroke 2006 37 10 2508 13 10.1161/01.STR.0000242290.01174.9e 16960091

